# Comparing feces collection methods for evaluating the apparent digestibility coefficient of brewers’ spent yeast in juvenile Atlantic salmon (*Salmo salar*)

**DOI:** 10.3389/fvets.2024.1449221

**Published:** 2024-08-14

**Authors:** Paola Orellana, Lorenzo Márquez, Alexander Ortloff, Joceline Ruiz, Patricio Dantagnan, Adrián J. Hernández

**Affiliations:** ^1^Doctorado en Ciencias Agropecuarias, Facultad de Recursos Naturales, Universidad Católica de Temuco, Temuco, Chile; ^2^Centro de Investigación, Innovación y Creación (CIIC-UCT), Facultad de Recursos Naturales, Universidad Católica de Temuco, Temuco, Chile; ^3^Departamento de Ciencias Veterinarias y Salud Pública, Facultad de Recursos Naturales, Universidad Católica de Temuco, Temuco, Chile; ^4^Núcleo de Investigación en Producción Alimentaria, Departamento de Ciencias Agropecuarias y Acuícolas, Facultad de Recursos Naturales, Universidad Católica de Temuco, Temuco, Chile

**Keywords:** microbial protein, digestibility of protein ingredients, diet replacement method, brewery wastes, salmonid aquaculture

## Abstract

Brewer’s spent yeast (BSY), derived from *Saccharomyces cerevisiae* used in beer production, is a valuable protein source for aquafeeds. Estimations of apparent digestibility coefficients (ADC) for nutrients in BSY are crucial for its inclusion in aquafeeds. ADC estimations for *Saccharomyces cerevisiae* protein in rainbow are hardly comparable from a methodological point of view, whereas the ADC estimations for BSY protein in Atlantic salmon are only based on stripped feces, which are known to produce underestimations. Therefore, new determinations of ADC of BSY nutrients are necessary for the inclusion of this ingredient in practical diets for salmonids. This study is focused on determining unbiased ADC values for protein and energy from BSY in juvenile *Salmo salar*. To reduce systematic biases, fecal samples were collected using stripping and decantation methods, which are known to produce under-and overestimations, respectively. 780 fish (25.16 ± 4.88 g) were stocked in six tanks. A reference diet (50% protein, 20% lipid, 1% Cr_2_O_3_) was provided to three tanks, and a test diet (70,30 reference diet to BSY) to the other three. ADC for BSY protein was 84.70 ± 1.04% (decantation) and 70.50 ± 4.03% (stripping). For gross energy, stripped feces yielded an ADC of 52.04 ± 5.30%, while decantation resulted in 63.80 ± 1.17%. Thus, ADC estimates were taken as the average of the stripping-value and the decantation-value, resulting in 77.6% for BSY crude protein, which is appreciably higher than previously measured values in *S. salar* fed undisrupted *S. cerevisiae*, and in 57.9% for gross energy.

## 1 Introduction

World aquaculture has experienced a remarkable growth, making it the fastest-growing animal food-production sector globally (1). Aquaculture production heavily relies on the use of formulated or balanced aquafeeds to ensure high yields and optimal efficiency (2). Aquaculture of feed-requiring species significantly exceeds that of non-feed-requiring species, reaching 73.1% of total production by 2022 (3). The use of marine ingredients for aquafeed production is considered to be one of the main constraints for this vital industry to continue its growth and achieve sustainable development. In order to address this problem, it has been proposed using new raw materials to cover fish nutrient requirements (4, 5). The search for new sources of protein derived from industrial wastes is promising for developing the circular economy in animal production, including aquaculture.

Microorganisms and microbially-derived ingredients are presently being tested as complements for the formulation of nutritionally efficient aquafeeds (6–9). These microorganisms can be cultured using residues supplied by various industries. Therefore, applying SCP as innovative feed ingredients would reduce the quantity of industrial wastes and provides a sustainable source of proteins for aquaculture. Single-cell proteins have desirable characteristics as a nutritional supplement and can be produced at any time of the year due to their independence from seasonal and climatic variations (10). They do not require large land areas for their cultivation, but can be obtained from well-known and well-studied technologies such as industrial fermentations. In addition to evaluating different single-cell proteins as ingredients for aquafeeds, it is important to direct efforts towards making spent microbial biomass useful for the bioeconomy (11). One such industrial by-product is brewer’s spent yeast (BSY), which possesses desirable features such as high nutrient availability, and predictable supply, so that it can be incorporated in aquafeeds (12).

The inclusion of non-conventional ingredients in animal feeds can be challenging due to the specific characteristics of their matrix composition, which can limit nutrient bioavailability (13). In particular, the accurate formulation of industrial aquafeeds necessitates unbiased estimations of apparent digestibility coefficients (ADC) of protein and energy in these new ingredients (14). Some studies have reported ADC values for proteins derived from brewer’s spent yeast, as well as for its primary component, the yeast *Saccharomyces cerevisiae*, in salmonid fish species. Chen et al. (15), Nazzaro et al. (16), Estévez et al. (17) evaluated protein ADC of BSY in rainbow trout (*Oncorhynchus mykiss*). Meanwhile, other authors have examined the protein ADC of various subproducts derived from bakery’s *Saccharomyces cerevisiae* in freshwater-reared Atlantic salmon (*Salmo salar*) and also in smolts (18, 19). However, significant methodological differences among authors indicate that the data lack comparability, posing challenges for aquafeed formulators seeking a practical ADC value from scientific literature. In particular: (i) the formula used to calculate the ADC of ingredients according to the diet replacement method was not always the corrected version of Forster (20, 21) modified by Bureau and Hua (22), (ii) the formulation of the reference diet was not constant in some of the experiments, and probably more important, (iii) feces collection methods varied among authors and each study included only one method of collection.

It is well known that the method used to collect fish feces can influence the estimation of nutrient ADCs. For instance, the Guelph/decantation method (23) tends to overestimate ADCs, while the abdominal stripping method (24) tends to underestimate them, particularly in salmonid species (25, 26). The accurate estimation of ADC values remains crucial for incorporating brewer’s spent yeast (as well as for any other single-cell-derived protein ingredient) into aquafeed formulas for salmonids. Whenever possible, employing multiple feces collection methods with contrasting biases is advisable to ensure more robust and reliable estimations.

For those reasons, this study aims to estimate ADC for the protein and energy of BSY in juvenile Atlantic salmon, while controlling for estimation biases due to the feces collection method. ADC values will be estimated following the diet replacement method, with two feces collection methods with opposing biases in the ADC estimation: decantation columns, and stripping.

## 2 Methods

The experimental diets and the digestibility trial were performed in the facilities of the Department of Agricultural and Aquaculture Sciences, Natural Resources Faculty, Catholic University of Temuco, Chile.

### 2.1 Experimental diets

Brewer’s spent yeast (BSY) was sourced from CCU Chile Company, located in the Araucanía Region. The BSY by-product was carefully dried at 45°C for 48 h and subsequently milled using an ultra-centrifugal mill equipped with a 120 μ mesh A basal or reference diet, and a test diet including 70% of the reference diet, and 30% of the yeast ingredient were formulated. The inclusion rate of BSY was set at 30% of BSY because this value is recommended by Glencross et al. (27), and it is frequently used by different authors when estimating ingredients’ ADCs through the diet-replacement method (18, 19, 28). The formulation and proximate composition of the reference diet and the test diet are presented in Table 1. All components were mixed according to the established formulation, and deionized water was gradually added until a consistent wet dough was formed. Chromic oxide was included as an indicator in the reference diet at 1%. The wet diets were pressed in a meat grinder through a 2-mm die and dried in a forced-air oven (Venticell 707, MMM Med centre, Munich, Germany) at 50°C for 6 h. After preparation, the diets were stored in sealed airtight containers at −20°C.

**Table 1:** Formulation and proximate composition (dry weight) of experimental diets used in the determination of apparent digestibility of nutrients in brewer’s spent yeast.

Ingredient (%)	Reference	BSY meal
Fish meal^1^	70.00	49.00
Spent Brewer’s yeast meal^2^	0	30.00
Cassava starch^3^	16.00	11.20
Fish oil^4^	12.00	8.40
Vitamin-mineral premix^5^	1.00	0.70
Cr_2_O_3_^6^	1.00	0.70
TOTAL	100	100
Proximate composition (%)		
Dry matter	97.00	95.70
Protein	52.20	52.50
Lipid	19.80	14.80
Fiber	1.00	1.10
Ash	12.30	10.80
Carbohydrates	14.80	20.80
Phosphorus	1.60	1.50
Cr_2_O_3_	1.10	0.80
Gross energy (MJ/Kg)	22.60	21.80

1 Chilean Jack Mackerel (*Trachurus murphyi*), Orizon S.A.

2 CCU Chile.

3 Almisur S.A., Paraguay.

4 Chilean Jack Mackerel (*Trachurus murphyi*) oil, Orizon S.A.

5 Salmofood Vitapro Chile.

6 Sigma-Aldrich.

### 2.2 Fish and experimental conditions

Atlantic salmon (*Salmo salar*) juveniles, with an average weight of 25.16 ± 4.88 g (mean ± SD), were obtained from Tocoihue farm (Chiloé, Los Lagos Region, Chile). A total number of 780 animals were stocked in 6 tanks. The reference diet was supplied to 3 randomly selected tanks, and the test diet was supplied to the remaining tanks. Therefore, each treatment was replicated three times (the replication unit was the tank). The fish were kept under a natural photoperiod (10 h light and 14 h dark, winter period) at a temperature of 12.90 ± 0.10°C, mean oxygen saturation of 80.20 ± 4.70%. The temperature and dissolved oxygen were monitored daily using a sensor (YSI-55a, Yellow Spring, Ohio, USA). Fish were acclimated for 5 days in the experimental units and fed the reference diet. Following acclimatization, each group of fish was manually fed their respective diets (reference and test diets) twice daily, comprising 2% of the biomass to assure feeding in excess, over a period of 34 days.

### 2.3 Sample collection and digestibility calculations

Feces were collected over the final 2 weeks of the trial using 50 ml centrifuge tubes adapted to decantation columns of each experimental unit, following the Guelph system (23). Additionally, fecal samples were obtained by stripping (24) at the conclusion of the trial. The collected samples were lyophilized (Alpha 1–4 LSC basic, Martin Christ, Osterode, Germany) and homogenized for biochemical analyses. Apparent digestibility coefficients (ADC) of total, nutrient and ingredient were calculated according to the standard method described by (20, 22, 27):

$$\mathrm{AD}C_{\mathrm{Total}}\;\left(\%\right)=100-\left(100\times \frac{\left|Cr_{2}O_{3}\right|\;\mathrm{diet}}{\left|Cr_{2}O_{3}\right|\;\mathrm{faeces}}\right)$$

$$\mathrm{AD}C_{\mathrm{Nutrient}}\;\left(\%\right)=100-\;\left(100\times \frac{\mathrm{Nutrient}\;\mathrm{faeces}\times \left|Cr_{2}O_{3}\right|\;\mathrm{diet}\;}{\mathrm{Nutrient}\;\mathrm{diet}\times \left|Cr_{2}O_{3}\right|\;\mathrm{faeces}\;}\right)$$

$$\mathrm{AD}C_{\mathrm{ingredient}}=\frac{\left(\left(\mathrm{AD}C_{\mathrm{test}}\times \mathrm{Nutrient}\;\mathrm{test}\right)-\left(\mathrm{AD}C_{\mathrm{basal}}\times \mathrm{Nutrient}\;\mathrm{basal}\times 0.7\right)\right)}{0.3\times \mathrm{Nutrient}\;\mathrm{ingredient}}$$

where Cr_2_O_3_ feed and Cr_2_O_3_ feces = chromium content of the diet and feces, respectively, and Nutrient feces and Nutrient feed = content of the nutritional parameter of concern (dry matter, protein, or energy) in the diet and feces, respectively.

### 2.4 Chemical analyses

The proximate composition of the ingredients, experimental diets and feces were analyzed according to procedures standardized by the Association of Official Analytical Chemists AOAC (29). The dry matter was determined by gravimetry at 105°C until constant weight, and the ash content was calculated by incinerating the samples in a muffle furnace at 550°C for 5 h. The determination of total nitrogen was performed using the Kjeldahl method in a heated digester (DK20, Velp Scientifica, Usmate, Italy) with an automatic distillation unit (UDK 149, Velp Scientifica, Usmate, Italy), and protein was calculated using the factor N x 6.25. The total lipid content was determined gravimetrically in a Soxhlet extraction apparatus that uses petroleum benzine with a boiling range of 40–60°C. The crude fibre was obtained using a fibre extractor (FIWE 6, Velp Scientifica, Usmate, Italy), the nitrogen-free extract (NFE) was calculated as: 100% − (crude protein + crude lipid + ash + crude fibre). Total phosphorus was determined by colorimetry using vanadate-molybdate (method 965.17). The energy content was measured using a calorimeter bomb (C2000 basic, IKA, Wilmington, USA) in isoperibolic mode at 25°C. The chromium oxide content in the diets and feces was estimated according to Furukawa and Tsukahara (30). All analytical work was performed in duplicate. The analysis of amino acids was conducted by a certified laboratory, (Instituto de Ciencia y Tecnología de los Alimentos-ICYTAL, at Universidad Austral in Valdivia, Chile) utilizing high-performance liquid chromatography.

### 2.5 Statistics and calculations

Data on the amino acid contents in 20 ingredients (6 brewer’s spent yeast byproducts, 9 *S. cerevisiae* ingredients not from brewery industries, and 5 fish meals as compositional references) were subjected to a multidimensional scaling (MDS) analysis based on the Euclidean distance. MDS distances were based on 15 amino acids expressed as g per kg of dry matter, g (kg DM)^−1^ (Trp, Cys, and Glu were not included in the MDS analysis because not all authors provided data about their contents; Asn and Gln were also excluded because the usual HPLC protocols do not provide data on these amino acids). Data on growth and zootechnical variables reported in the present work were analysed by *t*-test to compare the reference and the BSY diets. Data on nutrient ADC’s were similarly analysed to compare both methods of stool collection, decantation and stripping. Normality and homogeneity of variance were checked with Shapiro–Wilk and Bartlett tests, respectively. In the case of non-normality, the Welch test was performed. Analyses were performed using R Studio software, version 1.4.1106.

Practical ADC’s for nutrients contained in BSY were estimated as the arithmetic mean of the ADC values obtain by both methods of feces collection. In addition, maximum errors of ADC estimates were proposed as half the interval (absolute value) between the ADC value for decanted feces and the ADC value for stripped feces.

$$\begin{array}{l} \mathrm{AD}C_{\mathrm{Nut}}\;\mathrm{estimation}=\\ \frac{\left(\mathrm{AD}C_{\mathrm{Nut}}\;for\;\mathrm{decantation}\right)+\left(\mathrm{AD}C_{\mathrm{Nut}}\;for\;\mathrm{stripping}\right)}{2} \end{array}$$

$$\begin{array}{l} \mathrm{AD}C_{\mathrm{Nut}}\;\mathrm{error}=\\ \frac{\left(\mathrm{AD}C_{\mathrm{Nut}}\;for\;\mathrm{decantation}\right)-\left(\mathrm{AD}C_{\mathrm{Nut}}\;for\;\mathrm{stripping}\right)}{2} \end{array}$$

## 3 Results

Overall, zootechnical variables measured over the 34-day feeding period indicate some significant differences in fish growth (Table 2). The mean final wet weight was 47.91 and 43.17 g for the reference and test diets respectively, and there was a significant difference in the specific growth rate (SGR) between dietary groups (*p* = 0.002). There was also a significant difference in the hepato-somatic index (HSI) between dietary groups (*p* = 0.010), but the effect of BSY inclusion on the total feed intake per fish was unclear (*p* = 0.053). The difference in FCR between diets was not statistically significant, and no difference in mortality rate between the reference and the test diet was found.

**Table 2:** The effects of brewer’s spent yeast on growth and physique indexes of juvenile Atlantic salmon (*Salmo salar*) after 34 days.

	Diet								
	Reference				BSY-meal				*p* value
Initial weight (g)	25.16	±	0.01		25.15	±	0.01		0.736
Final weight (g)	47.91	±	0.51	a	43.17	±	0.44	b	0.002
Weight gain rate (%)^1^	90.42	±	2.06	a	71.62	±	1.83	b	0.002
Total feed intake (g/fish)^2^	18.25	±	0.49		15.48	±	0.39		0.053
Feed conversion ratio (FCR)^3^	0.81	±	0.02		0.92	±	0.08		0.137
Specific growth rate (SGR)^4^	1.89	±	0.03	a	1.59	±	0.03	b	0.002
Condition factor (K)^5^	1.09	±	0.00		1.08	±	0.01		0.175
Survival rate (%)^6^	98.72	±	1.60		94.62	±	2.77		0.236
Viscero-somatic index (VSI)^7^	9.11	±	1.37		10.21	±	0.25		0.244
Hepato-somatic index (HSI)^8^	1.18	±	0.14	a	1.64	±	0.10	b	0.010

Degrees of significance (*p*-value) were estimated with a *t*-student. Different letters indicate significant differences among groups.

1 Weight gain rate (WGR, %) = 100 × [final weight (g) − initial weight (g)]/initial weight (g).

2 Feed intake (FI, g/fish) = dry feed fed (g)/fish number.

3 Feed coefficient rate (FCR) = dry feed fed (g)/[final body weight (g) − initial body weight (g)].

4 Specific growth rate (SGR, %/day) = 100 × ({Ln [final body weight (g)] − Ln [initial body weight (g)]}/days).

5 Condition factor (K) = 100 × fish weight (g)/[body length (cm)]^3^.

6 Survival rate (SR, %) = 100 × (surviving fish number/total fish number).

7 Viscerosomatic index (VSI, %) = 100 × [visceral weight (g)/body weight (g)].

8 Hepatosomatic index (HSI, %) = 100 × [liver weigh (g)/body weight (g)].

**Figure 1:**
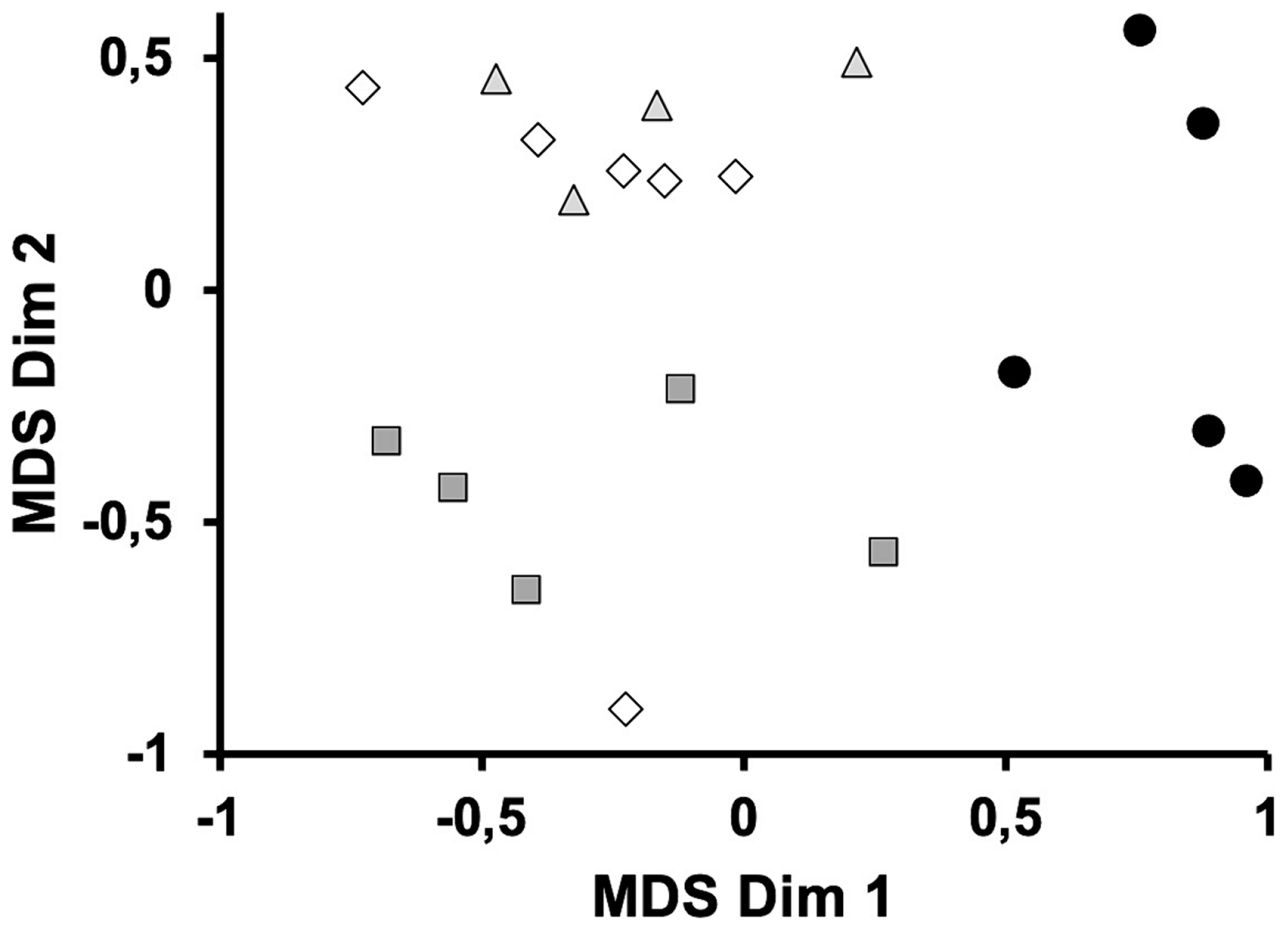
Bidimensional plot for an MDS analysis on the amino acid contents of protein ingredients. Black circles: fish meals; open diamonds: brewer’s spent yeasts; grey triangles: bakery yeasts *and S. cerevisiae* grown on residual sugars; grey squares: fast-growing *S. cerevisiae* grown under strong aeration (aerobiosis).

Regarding the MDS analysis of ingredients based on their aa content, MDS dimension 1 was positively associate with the content of nearly all amino acids, so that it can be interpreted as a proxy of ingredient protein content. On the other hand, MDS dimension 2 was positively associated with Glu, and negatively associated with the contents of His Tyr, and Arg, thus indicating that dimension 2 was more related to the relative composition of amino acids within an ingredient, than to the total content of protein. The bidimensional MDS plot (Figure 1) shows that ingredients with a similar organic origin tends to stand close to each other in the bidimensional MDS plot. Three groups were identified: fish meal ingredients (FM group), fast-growing *S. cerevisiae* cultured under strong aeration (ScA group) and, at last, brewer’s spent yeast and bakery yeast (BSY group). The FM group was characterized by its relatively high protein content (Dim 1), whereas ScA group and BSY group mainly differs in their relative amino acid compositions (Dim 2).

Table 3 shows the results of ADC of nutrients contained in BSY, considering the two methods of feces collection. ADC of dry matter (DM) was 58.75% according to the decantation method, while it was 40.41% for feces obtained by stripping. ADC of crude protein (CP) varied from 84.70% for feces collected by decantation to 70.50% for feces collected by stripping. When evaluating ADC of gross energy (GE), values of 52.04% were observed for the stripping method and 63.80% for the feces collected by decantation, with no significant statistical differences in this variable. Finally, ADC of the nitrogen-free extract (NFE) varied from 24.56% for decantation to 8.20% for stripping. A significant effect of the method of feces collection was observed for DM, CP, and NFE. Therefore, the practical estimates for ADC’s of DM, CP, GE, and NFE were 49.6, 77.6, 57.9, and 16.4%, respectively, and the maximal error of those estimations were 9.2, 7.1, 5.9, and 8.2%, respectively.

**Table 3:** Estimations of practical apparent digestibility coefficients (ADC) for nutrients in brewer’s spent yeast.

Nutrient	Analytical ADC		*p* value	ADC estimation	ADC error
	Guelph	Stripping			
DM^1^	58.75 ± 2.28^a^	40.41 ± 4.88^b^	0.028	49.6	9.2
CP^2^	84.70 ± 1.04^a^	70.50 ± 4.03^b^	0.038	77.6	7.1
GE^3^	63.80 ± 1.17	52.04 ± 5.30	0.068	57.9	5.9
NFE^4^	24.56 ± 1.63^a^	8.20 ± 4.64^b^	0.017	16.4	8.2

CP, crude protein; DM, dry matter; GE, gross energy; NFE, nitrogen-free extract.

## 4 Discussion

The average composition of BSY ingredients approximately contains 47,1 and 6% DM of crude protein, crude lipid, and ash, respectively (15, 16, 31–33). A MDS analysis showed that BSY ingredients, and non-brewery *S. cerevisiae* ingredients, generally contain less protein (related to MDS dim 1) than fish meals, but also that the variability in the amino acid distribution (related to MDS dim 2) among BSY samples is similar to the variability found among fishmeal samples. These compositional characteristics make BSY by-products good candidates to partially replace fish meal in formulated dies for salmonids (18, 34–37). However, there is still a clear necessity to publish new and more ADC determinations, in particular for the Atlantic salmon, for which only two estimations are currently available, both of them based on feces collected by stripping. Because no method of feces collection is free from systematic biases (25, 26), it seems very convenient to include at least two methods of collection with opposite biases when estimating ingredients’ ADCs, for example, decantation and stripping, which are the two ones more frequently reported in fish. This experimental design herein proposed makes it possible establishing a top limit (by decantation), and a bottom limit (by stripping) for the ADC estimation. However, this design is infrequently implemented. The situation could be alleviated if different authors had published ADC data based on different methods of feces collection to study the same ingredient in a given species. In that case, diet formulators can establish a sound interval for ADC that contains the true value. The midpoint of the interval can be considered as a reasonable estimation of ADC, and half the length of the interval can be used as an error term.

In the present work, the inclusion of the BSY byproduct in the test diet did not produce any signs of fish disease, nor it changed the survival rate. In addition, no significant effect was noted on the food conversion ratio (FCR). The BSY ingredient reduced fish consumption and growth rate by approx. 15%, however, fish continued showing an active feeding behaviour. The resulting relative feed intake per fish (1.3–1.5% BW) was comparable to ingestions reported by other authors working with Atlantic salmon of a similar size (35), and it was high enough for the reliability of a digestibility trial.

There are a few works reporting ADC values for BSY protein in *O. mykiss*, and at least two works showing ADC values for *S. cerevisiae* protein in juvenile *S. salar* (Supplementary material). According to those works, protein ADC of dried BSY varied between 57 and 75% in the rainbow trout (15–17, 36), while protein ADC of spray-dried or dried *S. cerevisiae* (not from breweries) were in the range 55–63% in the Atlantic salmon (18, 19). There is an additional work reporting an ADC of 86% for protein of bakery *S. cerevisiae* fed to *Salvelinus alpinus* (28). When the same ingredients were subjected to treatments that weaken cell walls, protein ADC increased to nearly 90%. At first sight, it is possible to think that those are enough data to estimate a value of protein ADC practical for formulators who want to include BSY or *S. cerevisiae* in salmonid diets. Indeed, they are not. The utility of data in the current literature is somewhat limited due to considerations related to the experimental design. For example, in the case of *O. mykiss*: (i) Rumsey et al. (36) used force-feeding techniques followed by the isolation of fish in metabolic chambers that impose highly stressing conditions on the animals, (ii) Cheng et al. (15) calculated ADC of protein BSY applying an equation without the necessary correction introduced by Foster (20) and Bureau and Hua (22), and (iii) Nazzaro et al. (16) and Estévez et al. (17) maintained the content of protein and lipids in the test diets, but the formula of the reference diet was not constant when mixed with the ingredient (27). Therefore, methodological variations made ADC estimations not comparable among authors in the case of *O. mykiss*.

In *Salmo salar*, two works testing *S. cerevisiae* biomass in diets for juvenile fish (18, 19) met the usual requirements for a digestibility trial: fish were subjected to low handling stress, the ADC equation took into account the correction proposed by Forster (20), and the formulation of the reference diet was constant when included in test diets. However, both works were based on collecting feces by the stripping method, thus both estimated protein ADC values are most probably underestimations (25, 26). This problem is particularly relevant when determining the digestibility of *S. cerevisiae* ingredients in salmonid species with the stripping method, since the probability of obtaining poorly digested materials can be increased by the enhancement of lysine transport (no data for other aa’s) in the distal intestine (37). ADC values for protein in dried *S. cerevisiae* obtained by Burr et al. (18) and Hansen et al. (19) were 63 and 51–56%, respectively. Although ADC estimations of *S. cerevisiae* protein with the decantation method in *S. salar* are not available, an estimation in *Salvelinus alpinus* is close to 86% (28). In the present study, ADC of BSY protein based on the stripping technique was close to 70%, clearly above the estimations by Burr et al. (18) and Hansen et al. (19). In addition, the estimation of protein ADC in *S. salar* based on the decantation technique was 85%, very close to the figure reported by Langeland et al. (28) in *S. alpinus*. The fact that the decantation technique produced higher ADC values than the stripping technique is in keeping with previous literature (25, 26). This result reinforces the consideration of decantation values as overestimations, and stripping values as underestimations, what is also supported by physiological studies (37).

Therefore, if the average of ADC values from both methods of collection of feces is taken as a minimally biased estimation, the experiment indicates that the ADC of BSY protein was 77.6% with an error equal to or below 7.1%. This value is appreciably higher than previously published values for protein ADC of undisrupted *S. cerevisiae* in juvenile *S. salar*. Following the same procedure, ADC of BSY gross energy (not previously reported for *S. salar*) was 57.9% with an error equal to or below 5.9%. The ADC estimations herein reported are probably more reliable than those previously published, because the application of decantation and stripping methods, in the same experiment, makes it possible to compensate the opposing biases and to calculate an error term. This experimental design implies more work because of the double determination of nutrient ADCs, but it provides more practical ADC estimations for diet formulators.

## 5 Conclusion

The ADC of protein and energy of a brewer’s spent yeast of Chilean origin were estimated to be 77.5 and 57.9%, respectively, in juvenile *Salmo salar*. This work underscores the importance of accurate estimation of nutrients ADC in novel ingredients such as BSY when formulating aquafeeds. The study highlights the challenges associated with inconsistent ADC estimations. The results indicate significant differences in ADC values between the two fecal collection methods, emphasizing the necessity of develop new approaches to obtain more precise estimations. The use of combined methods can yield a more accurate representation of the ADC values for novel feed ingredients like BSY. Overall, it is important to continue the studies that allow methodological repeatability in nutritional studies to ensure the reliability of ADC estimations for optimizing aquafeed formulations.

## Data Availability

The raw data supporting the conclusions of this article will be made available by the authors, without undue reservation.

## References

[1] Naylor RL Hardy RW Buschmann AH Bush SR Cao L Klinger DH. A 20-year retrospective review of global aquaculture. Nature. (2021) 591:551–63. doi: 10.1038/s41586-021-03308-6, PMID: 33762770

[2] Glencross BD Baily J Berntssen MH Hardy R MacKenzie S Tocher DR. Risk assessment of the use of alternative animal and plant raw material resources in aquaculture feeds. Rev Aquac. (2019) 12:703–58. doi: 10.1111/raq.12347

[3] FAO. The state of world fisheries and aquaculture 2024. Rome: Blue transformation in action (2024).

[4] Encarnação P. Functional feed additives in aquaculture feeds In: Nates SF, editor. Aquafeed formulation. Amsterdam: Academic Press (2016). 217–37.

[5] Vijayaram S Sun YZ Zuorro A Ghafarifarsani H van Doan H Hoseinifar SH. Bioactive immunostimulants as health-promoting feed additives in aquaculture: a review. Fish Shellfish Immun. (2022) 130:294–308. doi: 10.1016/j.fsi.2022.09.011, PMID: 36100067

[6] Agboola JO Lapena D Øverland M Arntzen MØ Mydland LT Hansen JØ. Yeast as a novel protein source-effect of species and autolysis on protein and amino acid digestibility in Atlantic salmon (*Salmo salar*). Aquaculture. (2022) 546:737312. doi: 10.1016/j.aquaculture.2021.737312

[7] Chen F Leng Y Lu Q Zhou W. The application of microalgae biomass and bio-products as aquafeed for aquaculture. Algal Res. (2021) 60:102541. doi: 10.1016/j.algal.2021.102541

[8] Shah MR Lutzu GA Alam A Sarker P Kabir MA Parsaeimehr A. Microalgae in aquafeeds for a sustainable aquaculture industry. J Appl Phycol. (2018) 30:197–213. doi: 10.1007/s10811-017-1234-z

[9] Trevi S Uren-Webster T Consuegra S de Leaniz G. Benefits of the microalgae *Spirulina* and *Schizochytrium* in fish nutrition: a meta-analysis. Sci Rep. (2023) 13:2208. doi: 10.1038/s41598-023-29183-x, PMID: 36750713 PMC9905068

[10] Mekonnen MM Hoekstra AY. Water footprint benchmarks for crop production: a first global assessment. Ecol Indic. (2014) 46:214–23. doi: 10.1016/j.ecolind.2014.06.013

[11] Stikane A Baumanis MR Muiznieks R Stalidzans E. Impact of waste as a substrate on biomass formation, and optimization of spent microbial biomass re-use by sustainable metabolic engineering. Fermentation. (2023) 9:531. doi: 10.3390/fermentation9060531

[12] Gokulakrishnan M Kumar R Ferosekhan S Siddaiah GM Nanda S Pillai BR. Bio-utilization of brewery waste (brewer's spent yeast) in global aquafeed production and its efficiency in replacing fishmeal: from a sustainability viewpoint. Aquaculture. (2023) 565:739161. doi: 10.1016/j.aquaculture.2022.739161

[13] Dawood MA Koshio S. Application of fermentation strategy in Aquafeed for sustainable aquaculture. Rev Aquac. (2020) 12:987–1002. doi: 10.1111/raq.12368

[14] Hardy RW Barrows FT. Diet formulation and manufacture In: Halver JE Hardy RW, editors. Fish nutrition. Amsterdam: Academic Press (2003). 505–600.

[15] Cheng ZJ Hardy RW Huige NJ. Apparent digestibility coefficients of nutrients in brewer's and rendered animal by-products for rainbow trout [*Oncorhynchus mykiss* (Walbaum)]. Aquac Res. (2004) 35:1–9. doi: 10.1111/j.1365-2109.2004.00941.x

[16] Nazzaro J San Martin D Perez-Vendrell AM Padrell L Iñarra B Orive M. Apparent digestibility coefficients of brewer's by-products used in feeds for rainbow trout (*Oncorhynchus mykiss*) and gilthead seabream (*Sparus aurata*). Aquaculture. (2021) 530:735796. doi: 10.1016/j.aquaculture.2020.735796

[17] Estévez A Padrell L Iñarra B Orive M San Martin D. Brewery by-products (yeast and spent grain) as protein sources in rainbow trout (*Oncorhynchus mykiss*) feeds. Front Mar Sci. (2022) 9:862020. doi: 10.3389/fmars.2022.862020

[18] Burr G Peterson B Pietrak M Sealey W Block S Bowzer J. Effect of PROPLEX DY and PROPLEX T on growth performance of Atlantic salmon smolts. Aquac Res. (2020) 51:4689–97. doi: 10.1111/are.14814

[19] Hansen JØ Lagos L Lei P Reveco-Urzua FE Morales-Lange B Hansen LD. Down-stream processing of baker's yeast (*Saccharomyces cerevisiae*) – effect on nutrient digestibility and immune response in Atlantic salmon (*Salmo salar*). Aquaculture. (2021) 530:735707. doi: 10.1016/j.aquaculture.2020.735707

[20] Forster I. A note on the method of calculating digestibility coefficients of nutrients provided by single ingredients to feeds of aquatic animals. Aquac Nutr. (1999) 5:143–5. doi: 10.1046/j.1365-2095.1999.00082.x

[21] Forster I.P. (1996). “A revised equation to calculate coefficients of digestibility for nutrients in feedstuffs for fish”, in *VII International Symposium on Nutrition and Feeding of Fish, ISFNF*. College Station, TX.

[22] Bureau DP Hua K. Letter to the editor of aquaculture. Aquaculture. (2006) 252:103–5. doi: 10.1016/j.aquaculture.2006.01.028

[23] Cho C.Y. Bayley H.S. Slinger S.J. (1975). “An automated fish respirometer for nutrition studies”, *Proc. 28th Ann. Meeting of Can. Conf. for Fish. Res.*, Vancouver, BC.

[24] Nose T. On the metabolic fecal nitrogen in young rainbow trout. Bull Japan Soc Sci Fis. (1967) 17:97–105.

[25] Hajen WE Beames RM Higgs DA Dosanjh BS. Digestibility of various feedstuffs by post-juvenile Chinook salmon (*Oncorhynchus tshawytscha*) in sea water. 1. Validation of technique. Aquaculture. (1993) 112:321–32. doi: 10.1016/0044-8486(93)90393-D

[26] Vandenberg GW de la Noüe J. Apparent digestibility comparison in rainbow trout (*Oncorhynchus mykiss*) assessed using three methods of feces collection and three digestibility markers. Aquac Nutr. (2001) 7:237–45. doi: 10.1046/j.1365-2095.2001.00181.x

[27] Glencross B Booth M Allan GL. A feed is only as good as its ingredients – a review of ingredient evaluation strategies for aquaculture feeds. Aquac Nutr. (2007) 13:17–34. doi: 10.1111/j.1365-2095.2007.00450.x

[28] Langeland M Vidakovic A Vielma J Lindberg JE Kiessling A Lundh T. Digestibility of microbial and mussel meal for Arctic charr (*Salvelinus alpinus*) and Eurasian perch (*Perca fluviatilis*). Aquac Nutr. (2016) 22:485–95. doi: 10.1111/anu.12268

[29] AOAC. Official methods of analysis. 18th ed. Gaitherburg: Association of Official Analytical Chemists International (2011).

[30] Furukawa A Tsukahara H. On the acid digestion method for the determination of chromic oxide as an index substance in the study of digestibility of fish feed. Nippon Suisan Gakkaishi. (1966) 32:502–6. doi: 10.2331/suisan.32.502

[31] Kim BG Liu Y Stein HH. Energy concentration and phosphorus digestibility in yeast products produced from the ethanol industry, and in brewers’ yeast, fish meal, and soybean meal fed to growing pigs. J Anim Sci. (2014) 92:5476–84. doi: 10.2527/jas.2013-7416, PMID: 25367516

[32] Pongpet J Ponchunchoovong S Payooha K. Partial replacement of fishmeal by brewer's yeast (*Saccharomyces cerevisiae*) in the diets of Thai Panga (*Pangasianodon hypophthalmus* × *Pangasius bocourti*). Aquac Nutr. (2016) 22:575–85. doi: 10.1111/anu.12280

[33] Prandi B Faccini A Lanbertini F Bencivenni M Jorba M van Droogenbroek B. Food wastes from agrifood industry as possible sources of proteins: a detailed molecular view on the composition of the nitrogen fraction, amino acid profile and racemisation degree of 39 food waste streams. Food Chem. (2019) 286:567–75. doi: 10.1016/j.foodchem.2019.01.166, PMID: 30827648

[34] Calberg H Lundh T Cheng K Pickova J Langton M Vázquez Gutiérrez JL. In search for protein sources: evaluating an alternative to the traditional fish feed for Arctic charr (*Salvelinus alpinus* L.). Aquaculture. (2018) 486:253–60. doi: 10.1016/j.aquaculture.2017.12.027

[35] Øverland M Karlsson A Mydland LT Romarheim OH Skrede A. Evaluation of *Candida utilis*, *Kluyveromyces marxianus* and *Saccharomyces cerevisiae* yeasts as protein sources in diets for Atlantic salmon (*Salmo salar*). Aquaculture. (2013) 402-403:1–7. doi: 10.1016/j.aquaculture.2013.03.016

[36] Rumsey GL Hughes SG Smith RR Kinsella JE Shetty KJ. Digestibility and energy values of intact, disrupted and extracts from brewer's dried yeast fed to rainbow trout (*Oncorhynchus mykiss*). Anim Feed Sci Technol. (1991) 33:185–93. doi: 10.1016/0377-8401(91)90059-2

[37] Vidakovic A Langeland M Sundh H Sundell K Olstorpe M Vielma J. Evaluation of growth performance and intestinal barrier function in Arctic Charr (*Salvelinus alpinus*) fed yeast (*Saccharomyces cerevisiae*), fungi (*Rhizopus oryzae*) and blue mussel (*Mytilus edulis*). Aquac Nutr. (2016) 22:1348–60. doi: 10.1111/anu.12344

